# Double inferior scleral flaps with intraoperative mitomycin-C for uveal effusion syndrome: a case report

**DOI:** 10.1097/RC9.0000000000000669

**Published:** 2026-07-08

**Authors:** Munirah Z. Aldofyan, Abdullah S. Alkharashi, Essam A. Osman, Faisal A. Alrashed, Abdulrhman F. Albloushi

**Affiliations:** Department of Ophthalmology, College of Medicine, King Saud University, Riyadh, Saudi Arabia

**Keywords:** exudative retinal detachment, mitomycin-C, nanophthalmos, scleral windows, uveal effusion syndrome

## Abstract

**Introduction and importance:**

Uveal effusion syndrome (UES) is a rare condition characterized by fluid accumulation in the suprachoroidal space and is often associated with scleral thickening and impaired transscleral outflow. The management of UES is challenging. We present a case of UES successfully managed with a modified surgical technique involving double scleral flaps with mitomycin-C.

**Case presentation:**

A 43-year-old man presented with sudden, painless, decreased vision in his left eye. Clinical examination and imaging revealed bilateral optic disc swelling, choroidal thickening, and inferior exudative retinal detachment in the affected eye. The axial lengths were short, suggestive of nanophthalmos. After an unsuccessful trial of high-dose oral corticosteroids, the patient underwent surgery involving two inferior scleral windows using double scleral flaps, central sclerotomy, and intraoperative application of mitomycin-C. Histopathology revealed a thickened sclera without inflammation. The effusion resolved completely within 6 months postoperatively, with no recurrence over a 2-year follow-up.

**Discussion:**

UES remains a surgical challenge, particularly in patients with eyes that have thick sclerae, where conventional drainage is ineffective. The aim of our approach was to reduce scleral resistance to fluid egress while preserving structural integrity. Long-term patency of the scleral windows was likely maintained by the use of mitomycin-C, which helped prevent fibrosis. Compared with previously described techniques, this modification is effective, safe, and structurally conservative.

**Conclusion:**

Double scleral flaps combined with mitomycin-C can be an effective surgical option for managing UES. The long-term resolution achieved in this case supports the value of this technique.

## Introduction

Uveal effusion syndrome (UES) is a rare ophthalmic disorder, with most available literature limited to isolated case reports and small case series^[^1–5^]^. It is characterized by the accumulation of fluid in the suprachoroidal space, leading to exudative detachments of the retina, choroid, and ciliary body. First described by Brockhurst in 1963 and initially considered idiopathic, UES has since been associated with nanophthalmos, scleral thickening, and abnormal transscleral outflow. A proposed mechanism is impaired transscleral protein and fluid movement, causing a buildup of protein-rich fluid in uveal tissues^[^1–4^]^.

HIGHLIGHTS

- Uveal effusion syndrome (UES) is a rare ophthalmic condition, and its management can be challenging.
- This case described a novel modified surgical technique for UES.
- Double scleral flaps with mitomycin-C are an effective option for managing UES.
- The long-term successful outcome highlights the importance of surgical intervention in UES.

This is particularly evident in eyes with abnormally thick or rigid sclerae, which act as a barrier to the normal egress of protein and fluid, raising hydrostatic pressure and producing uveal detachment. Surgical management, including choroidal drainage with sclerectomies or sclerostomies (scleral windows), remains the mainstay of treatment for patients resistant to medical therapy^[^5^]^.

In our case, we present a case of UES successfully managed with a modified scleral decompression technique involving two inferior double scleral flaps and adjunctive mitomycin-C. This report follows the SCARE guidelines^[^6^]^.

## Case presentation

**Figure 1.**
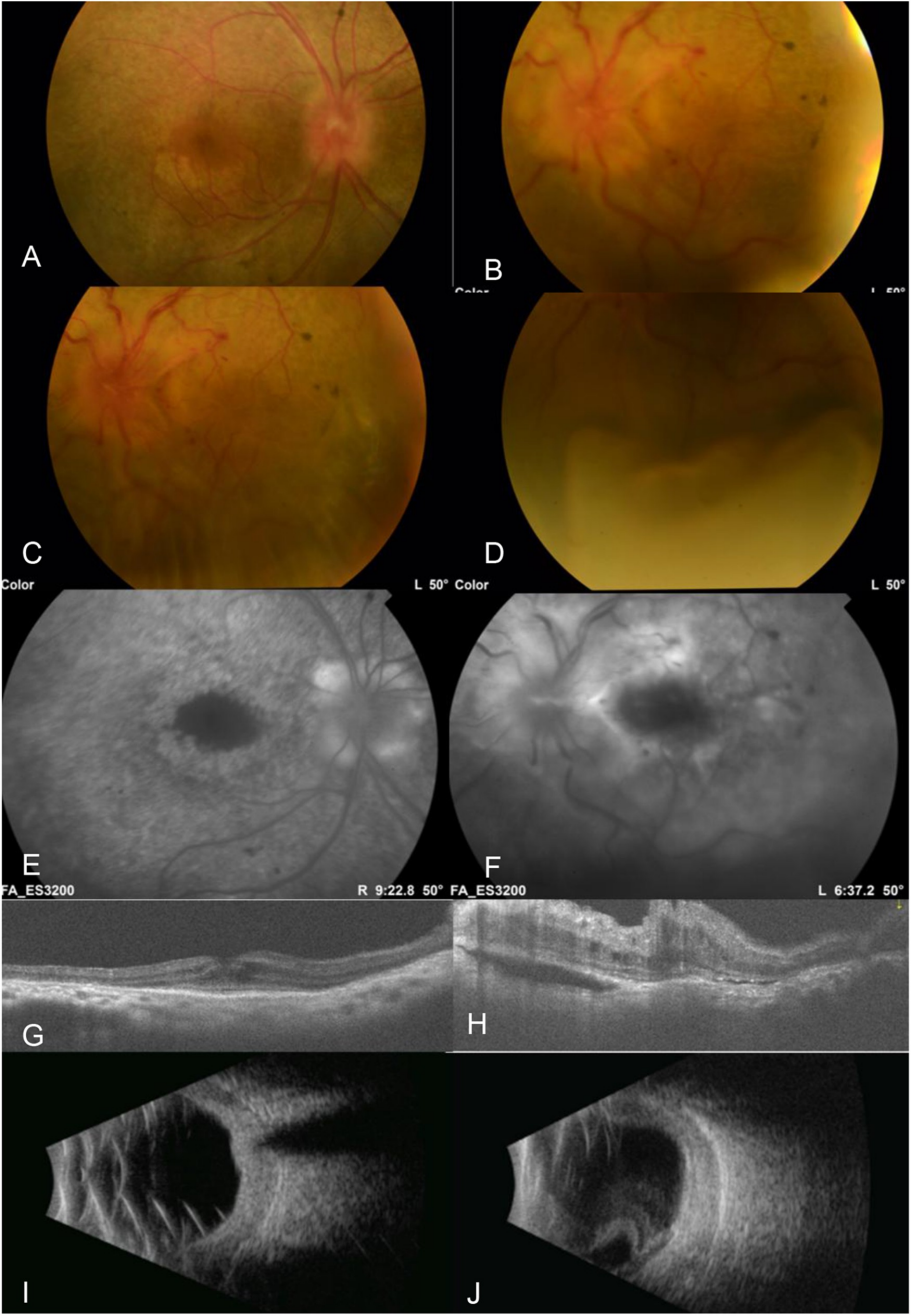
(A–C) Color fundus images showing bilateral optic disc swelling and macular thickening with peripheral pigmentary changes. The left eye exhibited more severe changes, including severe disc swelling. (D) Inferior exudative retinal detachment. (E–F) Fundus fluorescein angiography for both eyes–right (E)and left (F)–showing no signs of retinal or optic disc inflammation. (G–H) Spectral domain optical coherence tomography (SD-OCT) of both eyes showing severe subretinal and intraretinal fluids, as well as retinal wrinkles in the left eye (H). (I–J) B-scan ultrasonography showing bilateral retinochoroidal thickening and optic nerve head elevation in both eyes, with inferior retinal detachment extending from posterior to the equator and an intact superior retina in the left eye (J).

A 43-year-old man was referred for retinal detachment and disc swelling in his left eye, with a 10-day history of sudden, painless, curtain-like vision loss in the superior nasal field. His systemic review, social, drug, and family histories, as well as general examination, were unremarkable. Best-corrected visual acuity (BCVA) was 20/25 (right) and 20/60 (left); subjective refraction +9.00−2.00×150 (right) and +8.00−1.00×165 (left); intraocular pressure 18 mmHg (right) and 14 mmHg (left). Slit-lamp examination was unremarkable bilaterally. Dilated fundus examination showed bilateral optic disc swelling and macular thickening with mid-peripheral and peripheral pigmentary changes; the left eye showed bullous exudative inferior retinal detachment with more extensive disc swelling and pigmentary changes (Fig. 1 A–D). Fluorescein angiography showed no optic disc inflammation (Fig. 1 E–F).

SD-OCT revealed subretinal and intraretinal fluid, as well as retinal wrinkles (Fig. 1 G–H). B-scan ultrasonography showed bilateral retinochoroidal thickening and optic nerve head elevation, with inferior retinal detachment extending posteriorly to the equator and an intact superior retina in the left eye (Fig. 1 I–J).

A-scan axial lengths were 20.0 mm (right) and 19.46 mm (left). Ultrasound biomicroscopy (UBM) demonstrated 360° supraciliary effusion. A comprehensive laboratory work-up, including complete blood count, liver and renal function tests, erythrocyte sedimentation rate, C-reactive protein, QuantiFERON-TB Gold, purified protein derivative skin test, syphilis serology, and chest X-ray, was unremarkable. A diagnosis of UES was made. A short corticosteroid trial (oral prednisone 1 mg/kg [80 mg] daily for 1 week) produced no improvement, and surgery was pursued.

**Figure 2.**
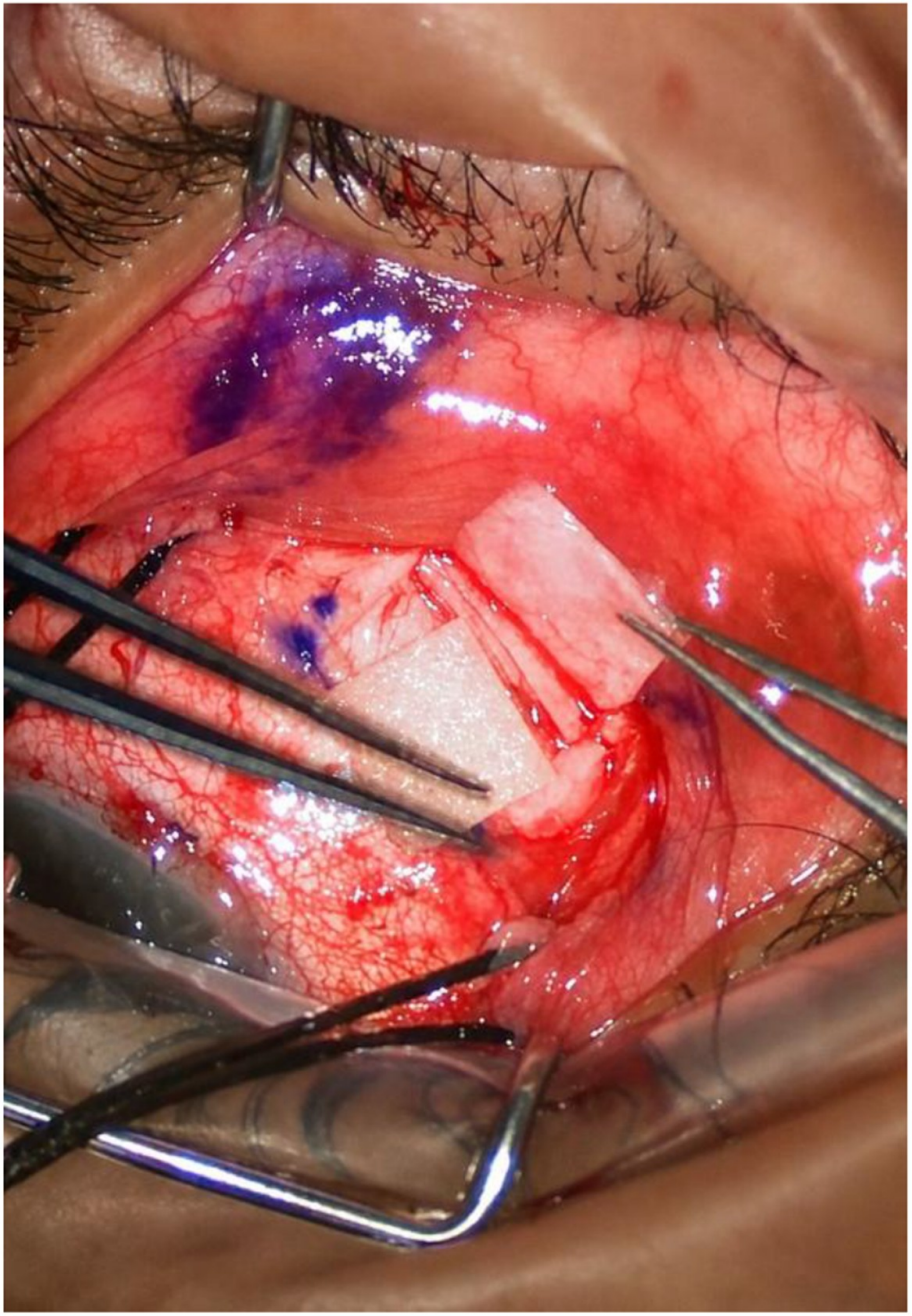
An intraoperative photograph demonstrates the thickness of the superficial scleral flap after the cutting of the deeper flap and during MMC application.

**Figure 3.**
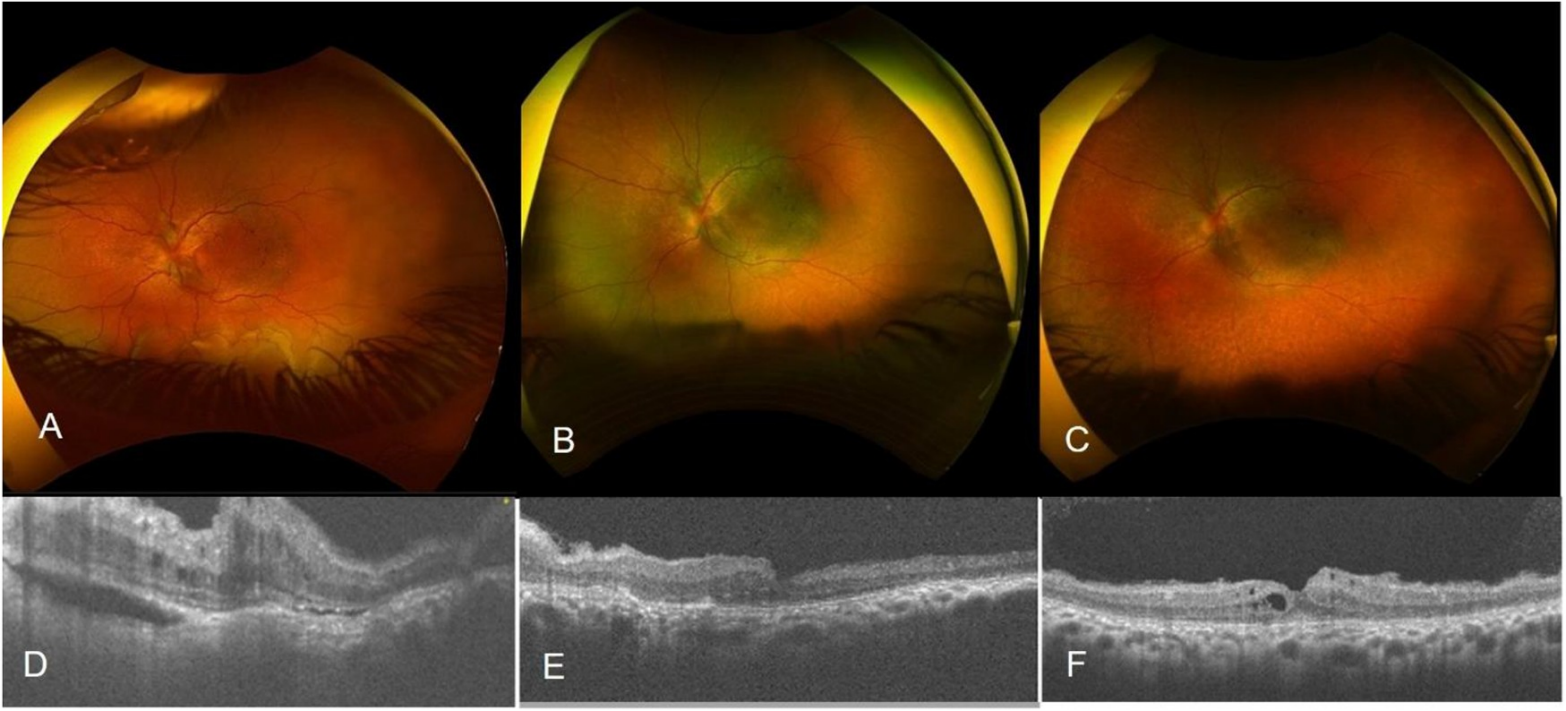
(A–F) Color fundus images and corresponding SD-OCTs of the left eye: (A, D) preoperatively, (B, E) 6 months postoperatively, and (C, F) 2 years postoperatively.

Two inferior scleral windows were created in the left eye using a modified technique: double scleral flaps with central sclerotomy and mitomycin-C 0.04% for 3 minutes. Although the sclera was thick intraoperatively (Fig. 2), histology of the excised sclera showed neither inflammation nor collagen disorganization. By 6 months, the uveal effusion had completely resolved, with total resolution of the exudative detachment sustained over 2 years of follow-up and a BCVA of 20/25 in both eyes. SD-OCT confirmed resolution of subretinal fluid with no thickening or choroidal folds (Fig. 3 A–F).

### Surgical technique

Under general anesthesia, two inferior scleral windows were created in the left eye. After a 180-degree inferior conjunctival peritomy, the inferior temporal and inferior nasal quadrants were dissected with curved Stevens scissors. The medial, lateral, and inferior recti were anchored with 4-0 silk sutures, and the sclera was exposed. A first scleral flap (5 × 5 mm, ~ 50% scleral thickness) was created 7 mm from the limbus, and a second, deeper 5 × 5 mm flap was raised as deep as possible up to the choroid, leaving a very thin scleral layer over it; the same was done in the other quadrant. Mitomycin-C 0.04% was applied for 3 minutes under the superficial flap, after which the deep flap was excised. A sclerotomy was made in the middle of the flap bed using a super-sharp blade and viscoelastic to push the choroid away, and a Kelly punch created an opening approximately 1.5 mm (about the size of the optic nerve); the inferior nasal quadrant was treated identically. The superficial flap was repositioned without suturing, the traction sutures were removed, and the conjunctiva was closed with 7-0 Vicryl (Supplemental Digital Content Video 1, available at: http://links.lww.com/IJSCR/A83).

## Discussion

The pathogenesis of UES is incompletely understood; proposed mechanisms include vortex vein obstruction, increased choroidal permeability, intrinsic choroidal abnormalities, and reduced scleral permeability. Histopathologic studies show that abnormal scleral thickness and collagen disorganization can impair transscleral protein and fluid movement, causing accumulation within the suprachoroidal and supraciliary spaces^[^2,4,7–11^]^. Uyama *et al* classified UES into three subtypes: type I (nanophthalmic eyes with markedly short axial lengths and high hyperopia), type II (non-nanophthalmic eyes with abnormal scleral thickness or composition), and type III (eyes without identifiable scleral abnormalities)^[^2^]^. In our patient, the short axial length (19–20 mm), hyperopic refractive status, intraoperative scleral thickening, and presence of supraciliary effusion were most consistent with type II UES.

UES most commonly affects middle-aged men, typically presenting with ciliochoroidal and serous non-rhegmatogenous retinal detachments, shifting subretinal fluid, and characteristic “leopard spot” pigmentary changes from chronic subretinal fluid^[^1,8,9,12^]^. In our patient, prominent pigmentary changes initially raised concern for a coexisting retinal dystrophy, an added diagnostic challenge, since chronic UES can itself cause secondary pigmentary alterations. Electrophysiologic and genetic testing were precluded by financial limitations; thus, although the clinical and intraoperative findings strongly supported type II UES, we could not definitively exclude a coexisting dystrophy, underscoring the complexity of atypical presentations and real-world diagnostic constraints.

Management remains challenging given the rarity and multifactorial pathophysiology of UES, with treatment aimed at preserving vision by improving transscleral drainage and promoting retinal and ciliochoroidal reattachment^[^8^]^. Described medical therapies include systemic corticosteroids, nonsteroidal anti-inflammatory agents, laser photocoagulation, and topical carbonic anhydrase inhibitors or prostaglandin analogs, which have variable effectiveness^[^12^]^.

Surgery is, therefore, central in persistent or refractory cases, and several decompression techniques have evolved. Brockhurst introduced scleral resection with vortex vein decompression in 1975, though it is technically demanding. Gass proposed quadrantic partial-thickness sclerectomies with central sclerostomies, avoiding vortex vein manipulation while achieving favorable outcomes^[^4,5^]^. Uyama *et al* refined this with subscleral sclerectomy, scleral flap elevation, and deep scleral excision to expose the choroid, with good results in eyes with scleral abnormalities^[^2^]^. More recently, Kong *et al* described full-thickness sclerotomies alone as a simpler option, though some patients needed repeat procedures^[^13^]^. In addition, adjunctive mitomycin-C has been reported to improve long-term scleral window patency by reducing postoperative fibrosis^[^12,14–17^]^.

Our case introduces a modification of these techniques, using two inferior scleral windows with double scleral flaps: a central sclerotomy was created, the deep flap excised, and the superficial flap left unsutured. Intraoperative mitomycin-C was applied to minimize fibrosis. Although mitomycin-C may theoretically increase the risk of scleral thinning or delayed healing, no MMC-related complications occurred.

This approach targets the underlying pathophysiology, reducing scleral resistance and enabling gravity-dependent suprachoroidal drainage while preserving structural integrity. Compared with traditional multi-quadrant procedures, it achieved effective, more targeted, and structurally conservative decompression, with complete effusion resolution, stable reattachment, and no recurrence over 2 years. Although larger studies are warranted, this modification may be a viable option in selected UES patients with thickened sclerae and impaired transscleral outflow.

## Conclusion

We report a case of UES successfully managed with double inferior scleral flaps combined with adjunctive mitomycin-C, resulting in the complete resolution of the effusion and stable retinal reattachment without recurrence over 2 years of follow-up. This case highlights how a targeted and structurally conservative scleral decompression approach may provide effective long-term control in selected patients with UES.

## Data Availability

The datasets used and/or analyzed during the current study are available from the corresponding author upon reasonable request.
